# Depression and Mood Changes in People with Parkinson’s Disease over Time: A 5-Year Follow-Up Study

**DOI:** 10.3390/jpm16070372

**Published:** 2026-07-10

**Authors:** Ángela Solleiro-Vidal, Diego Santos-García, Alfredo Puy Núñez, Teresa de Deus Fonticoba, Pablo Mir, Gracia Pons Pons, Juan García Caldentey, Nuria Caballol, Jorge Hernández Vara, Lydia López Manzanares, Bárbara Vives Pastor, Maria A. Ávila Rivera, Isabel González Aramburu, Rocío García-Ramos, Carmen Borrué, Julio Dotor García-Soto, María Álvarez Sauco, Iria Cabo, Guillermo González Ortega, COPPADIS Study Group

**Affiliations:** 1 Grupo de Investigación en Enfermedad de Parkinson y otros Trastornos del Movimiento, Instituto de Investigación Biomédica de A Coruña (INIBIC), 15006 A Coruña, Spain; 2 Complejo Hospitalario Universitario de A Coruña (CHUAC), 15006 A Coruña, Spain; 3 Hospital San Rafael, 15006 A Coruña, Spain; 4 Fundación Degen, 15006 A Coruña, Spain; 5 Networking Research Center on Neurodegenerative Diseases (CIBERNED), Instituto de Salud Carlos III, 28029 Madrid, Spain; 6 Complejo Hospitalario Universitario de Ferrol (CHUF), 15405 Ferrol, Spain; 7 Unidad de Trastornos del Movimiento, Servicio de Neurología, Instituto de Biomedicina de Sevilla, Hospital Universitario Virgen del Rocío/CSIC/Universidad de Sevilla, 41013 Seville, Spain; 8 Departamento de Medicina, Facultad de Medicina, Universidad de Sevilla, 41009 Seville, Spain; 9 Hospital Universitari Mutua de Terrassa, 08221 Terrassa, Spain; 10 Centro Neurológico Oms 42, 07003, Palma de Mallorca, Spain; 11 Complex Hospitalari Universitari Moisés Broggi, 08970 Sant Joan Despí, Spain; 12 Hospital Universitario Vall d’Hebron, 08035 Barcelona, Spain; 13 Hospital Universitario La Princesa, 28006 Madrid, Spain; 14 Hospital Universitario Son Espases, 07120 Palma de Mallorca, Spain; 15 Consorci Sanitari Integral, Hospital General de L’Hospitalet, 08906 L’Hospitalet de Llobregat, Spain; 16 Hospital Universitario Marqués de Valdecilla, 39008 Santander, Spain; 17 Instituto de Investigación Sanitaria Valdecilla (IDIVAL), 39011 Santander, Spain; 18 Hospital Clínico San Carlos, 28040 Madrid, Spain; 19 Hospital Infanta Sofía, 28702 Madrid, Spain; 20 Hospital Universitario Virgen Macarena, 41009 Sevilla, Spain; 21 Hospital General Universitario de Elche, 03203 Elche, Spain; 22 Complejo Hospitalario Universitario de Pontevedra (CHOP), 36161 Pontevedra, Spain; 23 Hospital Universitario de Móstoles, 28935 Madrid, Spain

**Keywords:** cohort, depression, mood, non-motor symptoms, Parkinson’s disease, prospective study

## Abstract

**Background and Objective:** Depression is frequent in Parkinson’s disease (PD), but it is unclear how mood changes and impacts patient’s quality of life (QoL) over time. Our objective was to analyze the frequency of depression and mood changes in people with PD (PwP) over 5 years of follow-up, comparing it with a control group, as well as its relationship with the patients’ QoL. **Patients and Methods:** PwP and healthy controls (HC) recruited from the COPPADIS cohort from January/2016 to November/2017 were included in this 5-year follow-up study. Mood was assessed by the Beck Depression Inventory II (BDI-II), and participants were classified as having major depression, minor depression, subthreshold depression, or non-depression at baseline and at 2, 4, and 5 years of follow-up. Correlation analysis and linear regression models were applied. **Results:** The BDI-II total score increased from 8.1 ± 6.2 at baseline to 10.3 ± 8.1 at the 5-year follow-up visit in PwP (*p* < 0.0001) but not in HC (from 4.1 ± 5.2 to 4.0 ± 5.7 [*p* = 0.896]). The prevalence of depression remained around 50–54% in the PwP group and 21–25% in the HC group throughout the follow-up period, but the mood state showed variability for each patient between visits. Patients’ QoL was associated with the depressive state throughout the entire follow-up period (*p* < 0.0001). Worsening QoL, sleep, longer disease duration, and an increase in neuropsychiatric symptoms were identified as independent factors associated with a worsening of mood over time in PwP (N = 348). **Conclusions:** Mood change over time in PwP is associated with QoL.

## 1. Introduction

Depression is one of the most frequent and disabling non-motor symptoms (NMS) in Parkinson’s disease (PD), and, in some patients, it may represent the main determinant of quality of life (QoL) [1,2,3]. In this context, NMS constitute a major source of disability that, in many cases, may outweigh that associated with the classical motor manifestations, significantly affecting both patients and caregivers. Among them, depression stands out as one of the most relevant neuropsychiatric features, alongside anxiety, fatigue, and pain [2].

The prevalence of depression in PD shows wide variability, reaching up to 90% depending on diagnostic criteria. However, it is estimated that approximately 35–40% of patients present clinically significant depressive symptoms, while 17–20% meet criteria for major depressive disorder [2,4,5]. Moreover, its incidence is approximately twice as high as in individuals without PD and can be up to ten times higher than that observed in the general population over 50 years of age [2]. Depressive symptoms may appear throughout the entire disease course, even preceding the clinical diagnosis. In this regard, population-based studies have shown that the risk of depression may be increased up to 8–10 years before PD diagnosis, and its frequency continues to rise thereafter [1,6]. In addition, a substantial proportion of patients present subclinical depressive symptoms, which are associated with an increased risk of progression to major depression, highlighting the importance of early identification [2]. Despite its high prevalence, depression in PD remains underdiagnosed, partly due to symptom overlap with the disease itself. Manifestations such as sleep disturbances, fatigue, or cognitive slowing may be attributed either to PD or to depression, complicating recognition and delaying treatment [2,4,7]. In addition, the presence of subclinical or milder depressive symptoms further contributes to diagnostic complexity and may hinder identification in clinical practice [2,4]. From a pathophysiological perspective, depression in PD is likely to have a multifactorial origin, involving alterations in multiple neurotransmitter systems—including dopaminergic, serotonergic, and noradrenergic pathways—together with dysfunction of fronto-limbic and cortico-striatal circuits, inflammatory processes, and neuroendocrine changes [2,6].

Depression in PD has consistently been associated with a more unfavorable clinical course, including greater motor, functional, and cognitive impairment, as well as increased mortality [1,2]. It is also associated with poorer QoL across all stages of the disease [8,9]. We observed in Spanish PD patients from the COPPADIS cohort that an increase of ≥5 points on the Beck Depression Inventory—II (BDI-II) after 2 years of follow-up multiplied the probability of presenting clinically significant health-related QoL impairment by 5 [10]. Even having subthreshold depression has been demonstrated to be associated with a worse QoL [11].

In spite of the available evidence, the longitudinal relationship between depression and the clinical progression of PD, as well as its differential impact according to symptom severity, including subclinical forms, has not been fully characterized [1]. Therefore, the aim of this study was to analyze the frequency and progression of depressive symptoms in people with PD (PwP) over a five-year follow-up period, compared with a healthy control group, and to assess their relationship with QoL.

## 2. Material and Methods

This observational, prospective, population-based, multicenter study included PwP and healthy controls (HC) recruited from 35 hospital centers across Spain, all belonging to the COPPADIS cohort [12]. Eligible patients had idiopathic PD according to the United Kingdom Parkinson’s Disease Society Brain Bank criteria, were aged 30–75 years, had no dementia (MMSE ≥ 26), and provided written informed consent. Exclusion criteria were inability to complete study assessments, disabling neurological or systemic comorbidities, chronic anemia and/or hyperuricemia, continuous levodopa or apomorphine infusion, deep brain stimulation, participation in another conflicting clinical study, or expected inability to complete long-term follow-up. Participants were recruited between January 2016 and November 2017. The study design corresponds to a five-year prospective multicenter longitudinal study aimed at analyzing PD progression in a Spanish population. The full methodology of the COPPADIS-2015 study has been previously described [13].

Participants were assessed at baseline (V0) and after 2 (V2), 4 (V4), and 5-year follow-up (V5). Mood was assessed using the BDI-II [14] a 21-item self-report questionnaire designed to evaluate the severity of depressive symptoms and for screening purposes. An item-based approach was used, selecting BDI-II items corresponding to DSM-IV depressive symptom domains: sadness (item 1), anhedonia (item 4), guilty feelings (item 5), suicidal thoughts or wishes (item 9), indecisiveness (item 13), loss of energy (item 15), sleep changes (item 16), irritability (item 17), and appetite changes (item 18). A symptom was considered present when the score for the corresponding item was ≥1. Based on the number and pattern of symptoms, participants were classified as follows [13]: major depression (≥5 symptoms, including sadness (item 1) and/or anhedonia (item 4), according to DSM-IV criteria), minor depression (2–4 symptoms, including item 1 and/or item 4), or subthreshold depression (2–4 symptoms without item 1 or item 4, according to Judd criteria) [15,16]. Participants not meeting these criteria were classified as non-depressed. This item-based classification has been previously applied in the COPPADIS cohort [13]. However, this approach does not constitute a clinical diagnosis and may be subject to misclassification, as the BDI-II is not a diagnostic instrument. QoL was assessed using three instruments: the 39-item Parkinson’s disease questionnaire (PDQ-39) [17]; PQ-10 [18]; and the EUROHIS-QOL 8-item index (EUROHIS-QOL8) [19]. The PDQ-39 is a PD-specific questionnaire comprising 39 items grouped into eight domains (mobility, activities of daily living, emotional well-being, stigma, social support, cognition, communication, and bodily discomfort), scored from 0 (never) to 4 (always) and referring to the previous four weeks. Scores are transformed into percentages and summarized as the PDQ-39 Summary Index (PDQ-39SI). The PQ-10 assesses overall perceived quality of life on a scale from 0 (worst) to 10 (best). The EUROHIS-QOL8, derived from the WHOQOL-BREF, includes 8 items (quality of life, health, energy, autonomy, self-esteem, social relationships, financial situation, and environment), scored from 0 to 5, with higher values indicating better QoL. The use of these three instruments was intended to capture complementary dimensions of quality of life, including PD specific health-related quality of life (PDQ-39), overall self-perceived quality of life (PQ-10), and generic quality of life and well-being (EUROHIS-QOL8).

Moreover, sociodemographic, clinical, comorbidity, and pharmacological treatment data were collected. The evaluation covered multiple clinical domains in addition to mood and QoL [13]: motor status (Hoehn and Yahr [H&Y], Unified Parkinson’s Disease Rating Scale [UPDRS-III and UPDRS-IV], Freezing of Gait Questionnaire [FOGQ]); non-motor symptoms (Non-Motor Symptoms Scale [NMSS], Parkinson’s Disease Sleep Scale [PDSS], Visual Analog Scale for Pain [VAS-Pain], Visual Analog Fatigue Scale [VAFS]); cognition (MMSE, Parkinson’s Disease Cognitive Rating Scale [PD-CRS]); neuropsychiatric symptoms (Neuropsychiatric Inventory [NPI], Questionnaire for Impulsive-Compulsive Disorders in Parkinson’s Disease-Rating Scale [QUIP-RS]); and disability (Schwab & England Activities of Daily Living Scale [ADLS]).

### 2.1. Statistical Analysis

Statistical analysis was performed using SPSS version 20.0 for Windows. Only participants with complete data for BDI-II were included. Quantitative variables were expressed as mean ± standard deviation (SD) or median [p25, p75], depending on their distribution. Categorical variables were expressed as frequencies and percentages. Normality was assessed using the Kolmogorov–Smirnov test. Group comparisons (major depression vs. minor depression vs. subthreshold depression vs. non-depression) were performed using the chi-square test or analysis of variance (ANOVA), as appropriate. To account for multiple comparisons in baseline between-group analyses, *p*-values were additionally adjusted using the Benjamini–Hochberg false discovery rate (FDR) procedure. The overall interpretation of the results was based on the FDR-adjusted analyses. Baseline comparisons were considered exploratory and descriptive in nature; therefore, post hoc pairwise comparisons were not conducted for any baseline analyses, and no adjustments for demographic or clinical covariates were applied. Changes in BDI-II scores between baseline (V0) and the 5-year follow-up (V5) were assessed using the Wilcoxon signed-rank test because the distribution of paired change scores did not meet the assumption of normality, as assessed by the Kolmogorov–Smirnov test. Correlations between the change in mood (BDI-II score) from V0 to V5 and the change in other continuous PD-related variables from V0 to V5 in PwP were analyzed using Pearson or Spearman correlation coefficients, depending on data distribution. Correlations were interpreted as weak (≤0.29), moderate (0.30–0.59), or strong (≥0.60).

To analyze factors associated with mood change, a multiple linear regression model was constructed using the change in BDI-II score from V0 to V5 (ΔBDI-II) as the dependent variable. Univariate analyses were first performed, and variables showing significant associations were included in the multivariate model. Following methodological recommendations, clinically relevant covariates were included: age, sex, disease duration, the change from V0 to V5 in the UPDRS-III (OFF), UPDRS-IV, FOGQ, PD-CRS, NMSS, PDSS, NPI, VAS-Pain, VASF-physical, VASF-mental, PDQ-39SI, EUROHIS-QOL8, and ADLS, and to be taking at V5 antidepressants, benzodiazepines, and antipsychotics. Model fit was assessed using the adjusted coefficient of determination (adjusted R^2^), and residual independence using the Durbin–Watson statistic. Results were expressed as β coefficients, 95% confidence intervals, and *p*-values. Multicollinearity problems were ruled out with Tolerance (a value below 0.20 indicates potential issues) and Variance Inflation Factor (VIF; VIF above 10 suggests severe multicollinearity).

### 2.2. Standard Protocol Approvals, Registrations, and Patient Consents

The study was approved by local and national research ethics committees in accordance with current regulations. All participants provided written informed consent prior to inclusion. The COPPADIS-2015 project was classified by the Spanish Agency of Medicines and Medical Devices (AEMPS) as a prospective post-authorization follow-up study (code: COH-PAK-2014-01).

### 2.3. Data Availability

The protocol and the statistical analysis plan are available on request. De-identified participant data are not available for legal and ethical reasons.

## 3. Results

**Figure 1:**
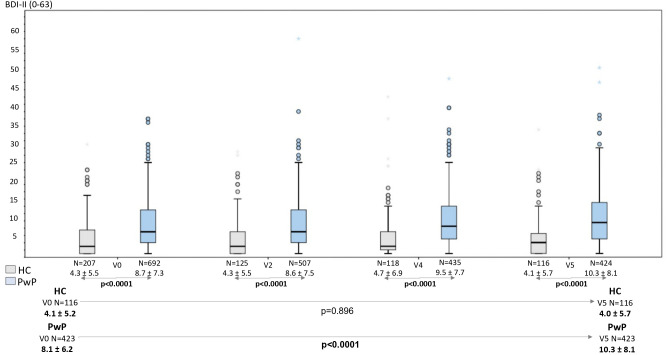
BDI-II scores at each visit throughout follow-up (V0, V2, V4, and V5) in PwP and HC from the COPPADIS cohort. Changes from V0 to V5 were analyzed using the Wilcoxon signed-rank test. HC, healthy controls; PwP, people with Parkinson’s disease. Circles indicate outliers, and asterisks indicate extreme outliers.

**Figure 2:**
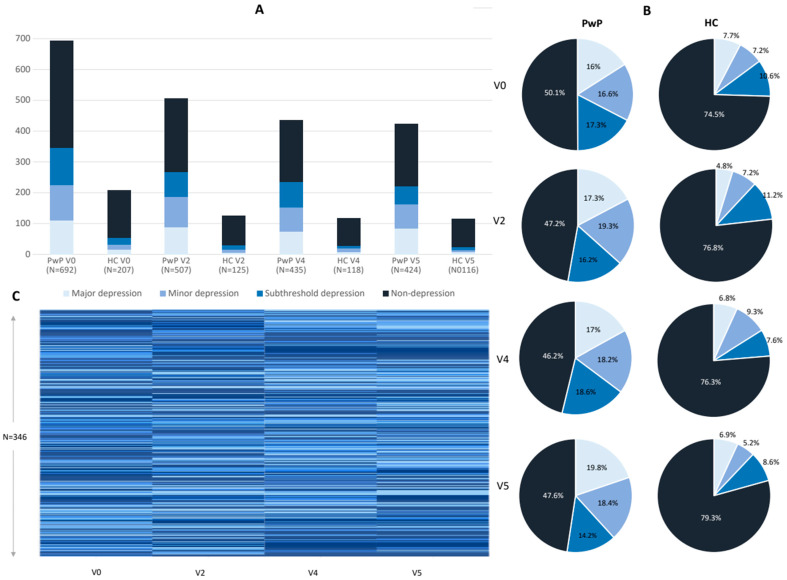
Number (N) of PwP and HC (**A**) and frequency (%) of PwP and HC (**B**) with major depression, minor depression, subthreshold depression, and non-depression at baseline (V0), 2-year (V2), 4-year (V4), and 5-year (V5) follow-up visits. (**C**) Mood state for each patient from the PwP group at each visit. HC, healthy controls; PwP, people with Parkinson’s disease.

A total of 692 PwP (62.6 ± 8.9 years old, 60.4% males) and 207 HC (61 ± 8.3 years old, 49.5% males) with complete data for BDI-II were recruited at baseline. In all visits (V0, V2, V4 and V5), the mean BDI-II score was significantly higher in the PwP group than in the HC group (*p* < 0.0001 for all visits) (Figure 1). The BDI-II total score increased from 8.1 ± 6.2 at baseline (V0) to 10.3 ± 8.1 at the 5-year follow-up visit (V5) in PwP (*p* < 0.0001) but not in HC (from 4.1 ± 5.2 to 4.0 ± 5.7 [*p* = 0.896]) (Figure 1). Depression (i.e., to have major depression, minor depression, or subthreshold depression) was significantly more frequent in PwP compared with HC at baseline (V0) and in all follow-up visits (V2, V4 and V5) (*p* < 0.0001 for all visits) (Figure 2A,B). Specifically, the prevalence of depression remained around 50–54% in the PwP group and 21–25% in the HC group throughout the follow-up period (Figure 2B).

At the individual level, longitudinal trajectory analysis in PwP (N = 346; PwP with data for mood stage classification in all visits) revealed marked variability in mood states, with frequent transitions between depression categories over time (Figure 2C). Only 17.1% of patients remained consistently non-depressed, while 34.4% of initially non-depressed individuals developed incident depression. Sustained remission was observed in only 8.4% of patients. Persistent depression was identified in 19.1%, whereas 18.2% exhibited a true relapse pattern following remission. Additionally, 9.5% showed a fluctuating course. Overall, 74.5% of the PwP cohort experienced depression at some point during follow-up (Figure 2C). Moreover, the frequency of PD patients who were receiving an antidepressant agent was 23.1%, 24%, 27.7% and 34.7% at V0, V2, V4 and V5, respectively.

At baseline, PwP with major depression tended to present a more severe motor affectation compared to other groups (Table 1). Specifically, they showed a higher score on the UPDRS-IV (*p* = 0.006) and FOGQ (*p* < 0.0001) compared with PwP with minor depression and subthreshold depression (Table 1). Regarding NMS, the NMS score was significantly higher in PwP with major depression (92.4 ± 51.4) compared to minor (56.2 ± 33.9), subthreshold (47.0 ± 32.3), and non-depressed PD patients (28.4 ± 24.7) (*p* < 0.0001). Similarly, cognitive performance showed lower PD-CRS total scores in PwP with major depression compared with the other groups (86.3 ± 14.7 vs. 87.7 ± 14.0 vs. 94.3 ± 16.0 vs. 94.5 ± 14.5; *p* < 0.0001), including the fronto-subcortical domain (*p* = 0.002) (Table 1). Neuropsychiatric symptoms (NPI), impulse control disorder (QUIP-RS), sleep quality (PDSS), pain (VAS-PAIN), and fatigue (VASF—physical and VASF—mental) followed a similar pattern, with scores showing greater impairment in PwP with major depression and more favorable profiles in non-depressed PwP (Table 1; *p* < 0.0001 for all analyses). These comparisons should be interpreted as descriptive, as no covariate adjustments were applied.

**Figure 3:**
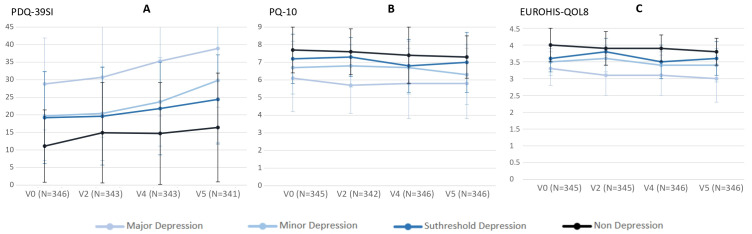
Mean and standard deviation on PDQ-39SI (**A**), PQ-10 (**B**), and EUROHIS-QOL 8 (**C**) at baseline (V0), 2-year (V2), 4-year (V4), and 5-year (V5) follow-up visits in PwP. The ANOVA test was used for comparison between all groups (major depression vs. minor depression vs. subthreshold depression vs. non-depression); *p* < 0.0001 for all analyses (**A**–**C**). EUROHIS-QOL8, EUROHIS-QOL 8-item index; PDQ-39SI, Parkinson’s Disease Questionnaire-39 item Summary Index.

According to the BDI-II total score, worsening depressive symptoms correlated with impairment in motor severity (UPDRS-III OFF; r = 0.186; *p* = 0.001), motor complications (UPDRS-IV; r = 0.104; *p* = 0.008), and freezing of gait (FOGQ; r = 0.222; *p* < 0.0001) (Table 2). Stronger associations were observed with an increase in NMS burden (NMSS, r = 0.460; *p* < 0.0001), neuropsychiatric symptoms (NPI; r = 0.461; *p* < 0.0001), physical (VASF–physical; r = 0.273; *p* < 0.0001) and mental fatigue (VASF–mental; r = 0.299; *p* < 0.0001), cognitive decline (PD-CRS; r = −0.240; *p* < 0.0001), and sleep impairment (PDSS; r = −0.274; *p* < 0.0001). Regarding QoL (PDQ-39SI; PQ-10; EUROHIS-QOL8) and disability (ADLS) at baseline, the scales again showed a worse QoL and less autonomy for activities of daily living in patients with major depression, with a pattern of less impairment the fewer depressive symptoms were present, with those without depression being the least affected (Table 1). These findings were similarly observed for health-related (PDQ-39SI) and global (PQ-10; EUROHIS-QOL8) QoL across all follow-up visits, with significant differences between groups in all analyses (*p* < 0.0001 for all analysis) (Figure 3).

Multivariate analysis identified several factors significantly associated with changes in depressive symptoms over 5 years in PwP (Table 3). Specifically, the change from V0 to V5 in the EUROHIS-QOL8 (r = −0.240; *p* = 0.001), NPI (r = 0.233; *p* = 0.001), PDSS (r = −0.187; *p* = 0.001), PDQ-39SI (r = 0.164; *p* = 0.036), and disease duration (r = 0.115; *p* = 0.041) were identified as independent factors associated with the change in the BDI-II from V0 to V5 after adjustment to covariates (Table 3). The multivariate model explained 44% of the variance in changes in depressive symptoms over 5 years (adjusted R^2^ = 0.44). The lowest value for Tolerance was 0.480 while the highest for VIF was 2.534, which ruled out collinearity problems.

## 4. Discussion

Our results showed that depressive symptoms are significantly more frequent in PwP than in HC and, additionally, tend to progress over time. In our cohort, approximately 50% of patients presented some form of depressive symptoms, with major depression between 17% and 19% throughout the 5-year follow-up. Importantly, the presence of depression in our study was significantly associated with greater clinical severity and functional impairment from baseline to the end of follow-up, highlighting its broad impact on disease progression.

The frequency of depression in our study coincides with what has been previously reported in the literature. Large meta-analyses and reviews report depressive disorders in about 30–40% of PwP, with major depressive disorder in about 14–19% [20,21]. When broader definitions or symptom scales are used, clinically significant depressive symptoms are seen in roughly 35–46% of patients [22,23]. Detecting the presence of depressive symptoms in PwP is important not only because it is common but also because its presence is associated with a worse clinical state. In our cohort, mood status was associated with the degree of clinical involvement in motor symptoms and NMS, as well as in QoL and disability, with PwP with major depression being the most affected and, at the other extreme, those without depression being the least affected. Across cross-sectional and longitudinal studies, depression in PD is clearly associated with more severe motor disease, higher disability, poorer QoL, and less favorable cognitive and survival [2,21,24,25,26,27,28,29]. Specifically, depressed PD patients have longer disease duration, higher motor scores (UPDRS-III), a higher H&Y stage, and lower activities of daily living scores than non-depressed patients [21,27]. Notably, patients with minor and subthreshold depression already exhibited a worse clinical profile compared to non-depressed individuals, suggesting that the association between depression and clinical impairment is present even at subclinical stages [11]. Consistent with this observation, previous studies have shown that even mild depressive symptoms may be associated with gait disturbances in early PD, reinforcing the clinical relevance of depressive symptoms across their full severity spectrum [30].

Regarding the NMS, patients with major depression showed a significantly higher burden, approximately three times greater than those without depression, while the minor and subthreshold groups displayed intermediate values. This is also conditioned by the fact that depressive symptoms are an aspect that is included in the evaluation (i.e., NMSS), and also because a more depressive mood can influence the perception of symptoms in general, including NMS as a whole or specific NMS such as pain or fatigue, as more disabling. Similarly, the burden of neuropsychiatric symptoms followed a comparable pattern, with a progressive increase according to depression severity, further supporting a close relationship between depression and overall neuropsychiatric burden. Previous studies found greater depressive severity in PD patients with pain [2,31] like in our cohort. Chronic pain may contribute to sleep disturbances, promoting fragmentation and reducing sleep quality [32]. In PD, this interaction is particularly relevant due to degeneration of the systems involved in the sleep–wake cycle, including noradrenergic, serotonergic, dopaminergic, and GABAergic pathways, contributing to the high prevalence of sleep disorders [32,33]. Accordingly, greater depressive severity was associated with poorer sleep quality, in line with evidence linking reduced sleep efficiency to neurobiological alterations and cognitive dysfunction, as we found, possibly mediated by changes in fronto-visual connectivity [34]. This relationship may reflect a bidirectional interaction between sleep and depression, partly mediated by GABAergic dysfunction and hyperactivation of the hypothalamic–pituitary–adrenal axis, forming a cycle of clinical deterioration [32,34,35].

In the present study, we observed how the change in mood in the long-term correlated with the worsening in many aspects of the disease, including both motor symptoms and NMS. In a large clinic cohort (N = 1214), Camerucci et al. observed that increasing depression severity was associated with worsening motor symptoms (UPDRS, H&Y), NMS, sleepiness, and poorer cognition [29]. Furthermore, depression is associated with greater disease progression and disability [26,36]. Regarding QoL, multiple studies and reviews identify depression as the primary driver of reduced QoL, often more impactful than motor severity itself [2,37,38,39,40]. In our study, worsening mood over the course of the disease was independently associated with longer disease duration and worsening sleep quality and an increase in neuropsychiatric symptoms, but also with a greater deterioration in global and health-related QoL. Understanding the patient’s mood is key, since in each follow-up visit it was associated with QoL; the greater the severity of depressive symptoms, the worse the QoL.

From a pathophysiological perspective, depression in PD can be understood as the result of a multifactorial model in which neurobiological mechanisms, clinical factors, and psychosocial elements interact [6] and as a manifestation of both the psychological response to the disease and the underlying neurodegenerative process itself [6,41]. Our findings align with this model, as greater overall impairment was associated with more severe depression, poorer QoL, and greater difficulties in activities of daily living, with these differences persisting throughout follow-up. Within this framework, motor and non-motor symptoms—including anxiety, apathy, pain, sleep disturbances, and cognitive decline—are closely related to depressive symptoms in a likely bidirectional manner. Greater clinical burden may increase vulnerability to depression through functional disability and reduced quality of life, while depressive symptoms may also influence the perception and clinical expression of disease manifestations, potentially contributing to a cycle of clinical deterioration [6,41]. Accordingly, depression in PD should be viewed not only as a marker of disease severity but also as part of a dynamic interplay between multiple clinical domains.

These clinical relationships may be underpinned by shared neurobiological mechanisms involving multiple neurotransmitter systems and neural circuits. Dopaminergic degeneration within the nigrostriatal and mesocorticolimbic pathways contributes not only to motor impairment but also to motivational and reward-processing deficits relevant to depression. In addition, serotonergic and noradrenergic dysfunction arising from brainstem nuclei has been implicated in mood regulation, sleep–wake control, pain perception, and fatigue [32,33,35]. Disruption of fronto-striatal and limbic networks, including prefrontal and cingulate regions, together with neuroinflammatory processes and hypothalamic–pituitary–adrenal axis dysregulation, may further contribute to both depressive symptoms and overall disease burden [34,35]. Collectively, these mechanisms provide a biological framework for the close and bidirectional relationship observed between depressive symptoms and clinical severity in Parkinson’s disease.

Despite therapeutic advances, remission rates in depression remain limited, with approximately 30% of patients not responding to treatment and high relapse rates [42]. In any case, it should be noted that while a high percentage of PD receive antidepressant treatment—between 1 in 3 and 4 patients in our overall cohort, depending on the follow-up visit—there are also patients meeting the criteria for major depression who do not receive it, reaching 50% at baseline in our cohort. Although previous studies estimate that 40–50% of patients with PD experience depressive symptoms during the disease course [43], our cohort showed a higher burden, with nearly 75% affected at some point during follow-up. Longitudinal analysis revealed that sustained remission was uncommon. Instead, 18.2% of patients exhibited a pattern of true relapse, while 19.1% showed persistent depression throughout follow-up, highlighting the recurrent and heterogeneous nature of depressive trajectories in PD.

The present study has some limitations. First, although it is a large, multicenter cohort with a five-year longitudinal follow-up, the observational nature of the design precludes establishing causal relationships between depression and the clinical progression of PD, limiting conclusions to associations. Second, depressive symptoms were assessed using clinical rating scales (BDI-II) rather than structured diagnostic interviews, which may introduce some heterogeneity in the classification of depression subtypes (major, minor, and subthreshold), as well as potential overlap with somatic symptoms inherent to PD. Furthermore, although multiple clinical variables were included and multivariate analyses were performed, the influence of unmeasured confounding factors cannot be excluded. These include social variables, specific psychopharmacological treatments, or therapeutic changes over time, all of which may have influenced the course of depressive symptoms. Baseline group comparisons were considered exploratory and descriptive in nature and were not adjusted for demographic (e.g., sex) or clinical covariates, and no post hoc pairwise comparisons were performed, which limits the interpretation of between-group differences at baseline. In addition, the potential loss of participants during follow-up and variability in sample size across visits may have introduced a degree of selection bias, potentially favoring patients with better clinical follow-up. Finally, although the study includes a comprehensive assessment of multiple clinical domains, some relevant constructs—such as anxiety and apathy—were not specifically analyzed in all models, which may limit a comprehensive characterization of the neuropsychiatric spectrum in Parkinson’s disease.

Overall, this study shows that depressive symptoms are highly prevalent in PD and follow a dynamic longitudinal pattern over time, characterized by an overall tendency toward worsening and marked inter-individual variability. Depression is consistently associated with a worse global clinical profile, including greater motor and non-motor severity, cognitive impairment, sleep disturbances, higher neuropsychiatric burden, poorer QoL, and increased functional dependency. This effect follows a gradient according to depressive severity and is already evident even in subclinical forms. Beyond this, the evolution of depressive symptoms appears to be modulated by multiple interrelated factors, such as QoL, sleep disturbances, and neuropsychiatric burden, reflecting a complex interaction between motor and non-motor domains. Taken together, these findings highlight the importance of early detection of depression in PD, even at mild or subclinical stages, as well as the need for a comprehensive and multidisciplinary clinical approach that integrates mental health assessment into routine neurological care, with the aim of optimizing overall disease progression and patients’ QoL.

## Figures and Tables

**Table 1:** Motor and non-motor symptoms, autonomy for activities of daily living, quality of life and other aspects related to PD in PwP at baseline (V0) according to mood state (N = 346).

	All Cohort (N = 346)	Major Depression (N = 46)	Minor Depression (N = 62)	Subthreshold Depression (N = 60)	Non Depression (N = 178)	*p*
Age	62.0 ± 8.7	61.5 ± 8.6	63.3 ± 7.9	62.7 ± 9.2	62.2 ± 8.7	0.399
Sex (males) (%)	57.7	39.1	54.8	61.7	62.1	**0.036** ^1^
Disease duration	5.4 ± 4.3	5.8 ± 4.6	4.7 ± 3.6	5.6 ± 5.2	5.4 ± 4.1	0.507
LEDD	545.3 ± 378.7	520.2 ± 370.1	522.4 ± 373.1	590.6 ± 348.3	544.5 ± 394.1	0.733
Motor phenotype (%):						0.074
-Tremor-dominant	49.7	39.1	51.6	53.3	50.6	
-PIGD	32.7	43.5	35.5	38.3	27	
-Indeterminate	17.6	17.4	12.9	8.4	22.4	
Hoehn & Yahr-OFF	2 [2,2]	2 [2,2.5]	2 [2,2]	2 [2,2]	2 [1.5,2]	**0.007**
-Stage from 3 to 5 (%)	7.4	18.2	8.2	6.9	4.3	0.097
UPDRS-III-OFF	21.9 ± 10.3	24.6 ± 9.9	21.6 ± 10.7	22.7 ± 10.9	21.0 ± 9.9	0.206
UPDRS-IV	1.9 ± 2.3	2.8 ± 2.2	2.2 ± 2.3	1.9 ± 2.3	1.6 ± 2.2	**0.006**
-Motor fluctuations (%)	33.5	50	37.1	33.3	28.1	**0.040** ^1^
FOGQ	3.6 ± 4.4	5.8 ± 4.4	3.9 ± 4.8	3.9 ± 4.4	2.8 ± 4.1	**<0.0001**
PD-CRS	92.2 ± 15.1	86.3 ± 14.7	87.7 ± 14.0	94.3 ± 16.0	94.5 ± 14.5	**<0.0001**
-FS sub-score	64.4 ± 13.8	59.3 ± 14.0	60.8 ± 12.4	66.3 ± 14.7	66.4 ± 13.4	**0.002**
-PC subscore	27.8 ± 3.3	27.0 ± 3.7	26.9 ± 4.4	28.1 ± 2.9	28.2 ± 2.8	**0.023**
NMSS	45.2 ± 38.8	92.4 ± 51.4	56.2 ± 33.9	47.0 ± 32.3	28.4 ± 24.7	**<0.0001**
BDI-II	8.2 ± 7.1	18.4 ± 6.9	11.5 ± 6.4	8.7 ± 4.5	3.6 ± 4.4	**<0.0001**
NPI *	5.8 ± 8.1	15.2 ± 12.1	7.8 ± 6.9	5.8 ± 6.8	2.6 ± 4.9	**<0.0001**
QUIP-RS	3.9 ± 7.8	5.5 ± 8.1	3.6 ± 6.9	7.6 ± 10.5	2.6 ± 4.9	**<0.0001**
PDSS	117.3 ± 24.6	100.2 ± 27.1	115.3 ± 19.4	119.3 ± 19.5	122.1 ± 25.1	**<0.0001**
VAS-PAIN	2.7 ± 2.9	4.3 ± 2.7	3.3 ± 3.3	2.7 ± 2.9	2.1 ± 2.8	**<0.0001**
VASF−physical	2.9 ± 2.7	5.3 ± 2.6	3.1 ± 2.6	3.6 ± 2.8	1.9 ± 2.3	**<0.0001**
VASF–mental	2.1 ± 2.5	4.6 ± 2.8	2.3 ± 2.4	2.3 ± 2.7	1.3 ± 2.0	**<0.0001**
ADLS	88.9 ± 10.1	83.3 ± 13.0	88.9 ± 10.1	88.7 ± 8.7	91.5 ± 8.5	**<0.0001**
PDQ-39SI	16.5 ± 13.2	28.9 ± 13.1	19.7 ± 12.2	19.3 ± 13.1	11.3 ± 10.4	**<0.0001**
EUROHIS-QOL8	3.8 ± 0.5	3.4 ± 0.6	3.5 ± 0.5	3.7 ± 0.5	4.0 ± 4.4	**<0.0001**
PQ-10	7.3 ± 1.6	6.1 ± 1.9	6.7 ± 1.5	7.3 ± 1.5	7.7 ± 1.3	**<0.0001**
Treatments (%):						
-Levodopa	68.8	80.4	64.5	70	66.9	0.286
-Dopamine agonist	71.4	58.7	64.5	75	75.8	0.067
- MAO-B inhibitor	75.4	65.2	66.1	80	79.8	**0.047** ^1^
- COMT inhibitor	18.8	16.9	19.4	20	18	0.546
-Amantadine	7.5	6.5	4.8	6.7	9	0.724
-Antidepressant	23.1	50	35.5	20	12.9	**<0.0001**
-Benzodiazepine	15.9	39.1	17.7	11.7	10.7	**<0.0001**
-Antipsychotic	1.2	2.2	0	1.7	1.1	0.734
-Analgesics	25.7	43.5	33.9	18.3	20.8	**0.003**

Note: The results represent percentages, mean ± SD or median [p25, p75]. Chi-squared and ANOVA tests (*p* value) were applied to compare between groups. Data about H&Y and UPDRS-III are during the OFF state (first thing in the morning without taking medication in the previous 12 h). False discovery rate (FDR) correction was performed using the Benjamini–Hochberg procedure. Motor phenotype, motor fluctuations, FOG, and falls were defined in the COPPADIS protocol according to the literature and scales used [14]. * Data represent the total score of the NPI (sum from domain A to J) informing about the subject, excluding the caregiver distress score. Not all information was collected for all variables, varying the range from 346 (most variables) to 290 (NPI). ADLS, Schwab and England Activities of daily living Scale); BDI-II, Beck Depression Inventory-II; FOGQ, Freezing of Gait Questionnaire; FOG, freezing of gait; NMSS, Non-Motor Symptoms Scale; NPI, Neuropsychiatric Inventory; PD, Parkinson’s disease; PDSS, Parkinson’s Disease Sleep Scale; PIGD, Postural Instability Gait Difficulty; QUIP-RS, Questionnaire for Impulsive-Compulsive Disorders in Parkinson’s Disease-Rating Scale; UPDRS, Unified Parkinson’s Disease Rating Scale; VAFS, Visual Analog Fatigue Scale; VAS-Pain, Visual Analog Scale-Pain. ^1^ Did not remain statistically significant after false discovery rate (FDR) correction using the Benjamini–Hochberg procedure.

**Table 2:** Correlation between the change in mood (BDI-II score) from V0 to V5 and the change in other PD-related variables from V0 to V5 in PwP (N = 346).

Change from V0 to V5	Change at V5 (V5–V0) (N = 346)	Correlation with Change in Mood (BDI-II) at V5 (V5–V0)	*p*
LEDD (mg/day)	+320.1 ± 422.3	−0.033	0.537
UPDRS-III (OFF)	+9.2 ± 14.4	0.186	**0.001**
UPDRS-IV	+1.7 ± 3.1	0.104	**0.008**
FOGQ	+2.8 ± 4.9	0.222	**<0.0001**
PD-CRS	−4.5 ± 17.2	−0.240	**<0.0001**
NMSS	+14.0 ± 40.4	0.460	**<0.0001**
BDI-II	+2.0 ± 7.3	N. A.	N.A.
PDSS	−0.6 ± 26.8	−0.274	**<0.0001**
QUIP-RS	−0.1 ± 9.5	−0.001	0.985
NPI	+3.2 ± 10.6	0.461	**<0.0001**
VAS-PAIN	+0.9 ± 3.5	0.095	0.079
VASF–physical	+1.1 ± 3.3	0.273	**<0.0001**
VASF–mental	+0.7 ± 3.4	0.299	**<0.0001**
PDQ-39SI	+7.2 ± 14.1	0.572	**<0.0001**
EUROHIS-QOL8	−1.6 ± 4.3	−0.491	**<0.0001**
PQ-10	0.4 ± 1.7	−0.246	**<0.0001**
ADLS	−10.3 ± 15.8	−0.375	**<0.0001**

Note: Spearman’s or Pearson rank correlation coefficient was applied. In bold, *p* < 0.05. ADLS, Schwab & England Activities of Daily Living Scale; BDI-II, Beck Depression Inventory-II; FOGQ, Freezing Of Gait Questionnaire; LEDD, levodopa equivalent daily dose; N.A., not applicable; NMSS, Non-Motor Symptoms Scale; NPI, Neuropsychiatric Inventory; PD-CRS, Parkinson’s Disease Cognitive Rating Scale; PDSS, Parkinson’s Disease Sleep Scale; QUIP-RS, Questionnaire for Impulsive-Compulsive Disorders in Parkinson’s Disease-Rating Scale; UPDRS, Unified Parkinson’s Disease Rating Scale; VAFS, Visual Analog Fatigue Scale; VAS-Pain, Visual Analog Scale-Pain.

**Table 3:** Factors associated with mood (BDI-II score) change from baseline (V0) to the 5-year follow-up visit (V5) (BDI-II_V5_–BDI-II_V0_) in PD patients (N = 346).

	β ^a^	95% CI ^a^	*p*	Durbin–Watson Test	Adjusted R^2^	β ^b^	95% CI ^b^	*p* ^b^
Change in the EUROHIS-QOL8	−0.491	−0.99–0.68	**<0.0001**	2.19	0.44	−0.240	−0.62–−0.17	**0.001**
Change in the NPI	0.461	0.25–0.39	**<0.0001**			0.233	0.07–0.25	**0.001**
Change in the PDSS	−0.274	−0.10–−0.05	**<0.0001**			−0.187	−0.09–−0.02	**0.001**
Change in the PDQ-39SI	0.572	0.25–0.34	**<0.0001**			0.164	0.01–0.17	**0.036**
Disease duration	0.092	−0.03–0.35	0.096			0.115	0.01–10.38	**0.041**

Note: Dependent variable: Change from V0 to V5 in the BDI-II total score. The β coefficient and 95% CI are shown. ^a^, univariate analysis; ^b^, multivariate analysis. Only data of variables significant in the multivariate analysis is shown. Factor included as covariates were age, sex, disease duration, the change from V0 to V5 in the UPDRS-III (OFF), UPDRS-IV, FOGQ, PD-CRS, NMSS, PDSS, NPI, VASF—physical, VASF—mental, PDQ-39SI, EUROHIS-QOL8 and S&E-ADLS, and to be taking at V5 antidepressants, benzodiazepines and antipsychotics. ADLS, Schwab & England Activities of Daily Living Scale; BDI-II, Beck Depression Inventory-II; FOGQ, Freezing Of Gait Questionnaire; NMSS, Non-Motor Symptoms Scale; NPI, Neuropsychiatric Inventory; PD-CRS, Parkinson’s Disease Cognitive Rating Scale; PDSS, Parkinson’s Disease Sleep Scale; UPDRS, Unified Parkinson’s Disease Rating Scale; VAFS, Visual Analog Fatigue Scale; VAS-Pain, Visual Analog Scale-Pain.

## Data Availability

The protocol, statistical analysis plan and unidentified participant data will be available on request.

## Abbreviations

ADLS, Schwab and England Activities of daily living Scale); BDI-II, Beck Depression Inventory-II; FOGQ, Freezing of Gait Questionnaire; FOG, freezing of gait; NMS, son-motor symptoms; NMSS, Non-Motor Symptoms Scale; NPI, Neuropsychiatric Inventory; PD, Parkinson’s disease; PDSS, Parkinson’s Disease Sleep Scale; PIGD, Postural Instability Gait Difficulty; PwP, people with Parkinson’s disease; QoL, quality of life; QUIP-RS, Questionnaire for Impulsive-Compulsive Disorders in Parkinson’s Disease-Rating Scale; UPDRS, Unified Parkinson’s Disease Rating Scale; VAFS, Visual Analog Fatigue Scale; VAS-Pain, Visual Analog Scale-Pain.

## Appendix A. COPPADIS STUDY GROUP

Adarmes-Gómez AD, Aldaz A, Almarcha Menargues M, Almeria Vivó M, Alonso Losada MG, Alonso Cánovas A, Alonso Redondo R, Álvarez Sauco M, Ascunce Vidondo A, Ávila MA, Borrue C, Cabello González C, Cabo López I, Caballol N, Carabajal Pendón E, Carrillo F, Casas E, Castellano Guerrero AM, Castejón Carbó J, Clavero P, Cots Foraster A, Cubo E, de Deus Fonticoba T, Díaz Galván P, Dotor García-Soto J, Escalante S, Estelrich Peyret E, Fernández A, Franco Rosado P, Gámez P, García Caldentey J, García-Ramos R, Gastón I, Gómez Garre P, González Aloy J, González Aramburu I, González García B, González Palmás MJ, Golpe Díaz A, Grau Solá M, Guardia Valls G, Hernández Vara J, Infante J, Jesús S, Kulisevsky J, Kurtis M, Labandeira C, Lacruz F, Legarda I, López Ariztegui N, López Domínguez D, López Manzanares L, López Valdés E, Lorenzo Barreto P, Lucas del Pozo S, Luque Ambrosiani AC, Macías Y, Macías García D, Mandrá MA, Mata M, Martínez Castrillo JC, Menéndez González M, Mir P, Miranda Santiago J, Morales Casado MI, Muñoz Delgado L, Muro García I, Novo Ponte S, Ojeda Lepe E, Ordás C, Pareés I, Pascual-Sedano B, Peral Quirós A, Pérez Calvo C, Pérez Noguera R, Planas-Ballvé A, Pons Pons G, Prats MA, Puig-Daví A, Puy Núñez A, Ribacoba C, Rivera Sánchez M, Rodríguez Pérez AB, Roldán F, Roson M, Ruíz Martínez J, San Eufrasio Martínez M, Sánchez Alonso P, Sánchez Díez G, Sánchez Martín ME, Sánchez Pelaez MV, Santacruz P, Santos García D, Seijo M, Sierra Peña M, Solano Vila B, Solares C, Solleiro Vidal A, Suárez Fuentes N, Tabar Comellas G, Torres Moral A, Valero C, Valero García, MF, Vela L, Vives Pastor B.

**Contribution**	**Role**	**Location**	**Name (Last Name, First Name)**
Evaluation of participants and/or data management	Site investigator	Hospital Universitario Virgen del Rocío, Sevilla, Spain	Adarmes-Gómez, Astrid Daniela adarmes-ibis@us.es
Evaluation of participants and/or data management	Site investigator	Hospital Clínico San Carlos, Madrid, Spain	Aldaz, Ana ana12aldaz@gmail.com
Evaluation of participants and/or data management	Site investigador	Hospital Ruber Internacional, Madrid, Spain	Almarcha Menargues, Marisa mlalmarcha@neurologiaclinica.es
Neuropsychologist; evaluation of participants	Site investigator	Hospital Universitari Mutua de Terrassa, Terrassa, Barcelona, Spain	Almeria Vivó, Marta malmeria@mutuaterrassa.cat
Coordination at the center Evaluation of participants and/or data management	Site investigator/PI	Hospital Álvaro Cunqueiro, Complejo Hospitalario Universitario de Vigo (CHUVI), Vigo, Spain	Alonso Losada, Maria Gema gemavarita@gmail.com
Evaluation of participants and/or data management	Site investigator	Hospital Universitario Ramón y Cajal, Madrid, Spain	Alonso Cánovas, Araceli aracelialonsocanovas@gmail.com
Coordination at the center Evaluation of participants and/or data management	Site investigator/PI	Hospital Universitario Lucus Augusti (HULA), Lugo, Spain	Alonso Redondo, Ruben alonso_1408@hotmail.es
Coordination at the center Evaluation of participants and/or data management	Site investigator/PI	Hospital General Universitario de Elche, Elche, Spain	Álvarez Sauco, María mariaalsa@hotmail.com
Evaluation of participants and/or data management	Site investigator	Complejo Hospitalario de Navarra, Pamplona, Spain	Ascunce Vidondo, Arancha arancha.ascunce.vidondo@navarra.es
Coordination at the center Evaluation of participants and/or data management	Site investigator/PI	Consorci Sanitari Integral, Hospital General de L’Hospitalet, L’Hospitalet de Llobregat, Barcelona, Spain	Ávila Rivera, Maria Asunción asuncion.avila@sanitatintegral.org
Coordination at the center Evaluation of participants and/or data management	Site investigator/PI	Hospital Infanta Sofía, Madrid, Spain	Borrué, Carmen carmenborrue@hotmail.com
Scheduling of evaluations	Site investigator	Complejo Hospitalario de Navarra, Pamplona, Spain	Cabello González, Carolina karol.cabello.gonzalez@navarra.es
Coordination at the center Evaluation of participants and/or data management	Site investigator/PI	Complejo Hospitalario Universitario de Pontevedra (CHOP), Pontevedra, Spain	Cabo López, Iria icabol@yahoo.es
Coordination at the center Evaluation of participants and/or data management	Site investigator/PI	Complex Hospitalari Universitari Moisés Broggi, Sant Joan Despí, Barcelona, Spain.	Caballol, Nuria nuriacaballol@hotmail.com
Evaluation of participants and/or data management	Site investigator (from MAR/23)	Hospital La Princesa, Madrid, Spain	Carabajal Pendón, Estefanía fanicarabajal@gmail.com
Evaluation of participants and/or data management	Site investigator	Hospital Universitario Virgen del Rocío, Sevilla, Spain	Carrillo, Fátima fmcarrillog@gmail.com
Evaluation of participants and/or data management	Site investigator	Hospital La Princesa, Madrid, Spain	Casas, Elena elena.caspe@gmail.com
Neuropsychologist; evaluation of participants	Site investigator	Hospital Universitari Mutua de Terrassa, Terrassa, Barcelona, Spain	Castejón Carbó, Judith jcastejon@mutuaterrassa.cat
Evaluation of participants and/or data management	Site investigator	Hospital Universitario Virgen del Rocío, Sevilla, Spain	Castellano Guerrero, Ana María acastellano-ibis@us.es
Evaluation of participants and/or data management	Site investigator	Complejo Hospitalario de Navarra, Pamplona, Spain	Clavero, Pedro p.clavero@hotmail.com
Evaluation of participants and/or data management	Site investigator	Institut d’Assistència Sanitària (IAS)-Instituí Cátala de la Salud. Girona, Spain	Cots Foraster, Anna acotsforaster@gmail.com
Coordination at the center Evaluation of participants and/or data management	Site investigator/PI	Complejo Asistencial Universitario de Burgos, Burgos, Spain	Cubo, Esther esthercubo@gmail.com
Nurse study coordinator Evaluation of participants and/or data management	Site investigator	Complejo Hospitalario Universitario de Ferrol (CHUF), Ferrol, A Coruña, Spain	De Deus Fonticoba, Teresa terecorreomovil@gmail.com
Neuroimaging studies	Site investigator	Hospital Universitario Virgen del Rocío, Sevilla, Spain	Díaz Galván, Patricia pdiaz-ibis@us.es
Evaluation of participants and/or data management	Site investigator/PI	Hospital Universitario Virgen Macarena, Sevilla, Spain	Dotor García-Soto, Julio juliodotor@gmail.com
Coordination at the center Evaluation of participants and/or data management	Site investigator/PI	Hospital Universitari de Tortosa Verge de la Cinta, Tortosa, Tarragona, Spain	Escalante, Sonia sescalant@yahoo.es
Evaluation of participants and/or data management	Site investigator	Institut d’Assistència Sanitària (IAS)—Instituí Cátala de la Salud. Girona, Spain	Estelrich Peyret, Elena elenaestelrich@gmail.com
Evaluation of participants and/or data management	Site investigator	Hospital Clínico San Carlos, Madrid, Spain	Fernández, Ana ana93fr@gmail.com
Neuroimaging studies	Site investigator	Hospital Universitario Virgen del Rocío, Sevilla, Spain	Franco Rosado, Pablo pfranco-ibis@us.es
Evaluation of participants and/or data management	Site investigator	Complejo Asistencial Universitario de Burgos, Burgos, Spain	Gámez, Pedro pgamezb@saludcastillayleon.es
Coordination at the center Evaluation of participants and/or data management	Site investigator/PI	Centro Neurológico Oms 42, Palma de Mallorca, Spain	García Caldentey, Juan juangcaldentey@hotmail.com
Coordination at the center Evaluation of participants and/or data management	Site investigator/PI	Hospital Clínico San Carlos, Madrid, Spain	García-Ramos, Rocío garciaramosg@yahoo.es
Coordination at the center Evaluation of participants and/or data management	Site investigator/PI	Complejo Hospitalario de Navarra, Pamplona, Spain	Gastón, Itziar itziar.gaston.zubimendi@cfnavarra.es
Genetic studies coordination	Site investigator	Hospital Universitario Virgen del Rocío, Sevilla, Spain	Gómez Garre, Pilar mgomez-ibis@us.es
Evaluation of participants and/or data management	Site investigator	Institut d’Assistència Sanitària (IAS)—Instituí Cátala de la Salud. Girona, Spain	González Aloy, Javier
Evaluation of participants and/or data management	Site investigator	Hospital Universitario Marqués de Valdecilla, Santander, Spain	González Aramburu, Isabel isagaramburu@gmail.com
Nurse study coordinator	Site investigator	Hospital La Princesa, Madrid, Spain	González García, Beatriz beaggmovil@gmail.com
Database gestion and coordination	Site investigator	Hospital Universitario de Móstoles, Madrid, Spain	González Ortega, Guillermo guilleglez93@gmail.com
Evaluation of participants and/or data management	Site investigator	Complejo Hospitalario Universitario de Pontevedra (CHOP), Pontevedra, Spain	González Palmás, Maria Josefa maria.josefa.gonzalez.palmas@sergas.es
Evaluation of participants and/or data management	Site investigator	Complex Hospitalari Universitari Moisés Broggi, Sant Joan Despí, Barcelona, Spain.	Grau Solá, Mireia mireia.grau@csi.cat
Evaluation of participants and/or data management; study coordinador	Site investigator	Hospital Universitari Mutua de Terrassa, Terrassa, Barcelona, Spain	Guardia Valls, Gemma gguardia@mutuaterrassa.cat
Coordination at the center Evaluation of participants and/or data management	Site investigator/PI	Hospital Universitario Vall d’Hebron, Barcelona, Spain	Hernández Vara, Jorge hernandezvarajorge76@gmail.com
Coordination at the center Evaluation of participants and/or data management	Site investigator/PI	Hospital Universitario Marqués de Valdecilla, Santander, Spain	Infante, Jon jinfante@humv.es
Evaluation of participants and/or data management	Site investigator	Hospital Universitario Virgen del Rocío, Sevilla, Spain	Jesús, Silvia smaestre-ibis@us.es
Coordination at the center Evaluation of participants and/or data management	Site investigator/PI	Hospital de Sant Pau, Barcelona, Spain	Kulisevsky, Jaime JKulisevsky@santpau.cat
Coordination at the center Evaluation of participants and/or data management	Site investigator/PI	Hospital Ruber Internacional, Madrid, Spain	Kurtis, Mónica mkurtis@ruberinternacional.es
Evaluation of participants and/or data management	Site investigator	CHUO (Complexo Hospitalario Universitario de Ourense), Ourense, Spain	Labandeira, Camen carmen.labandeira@hotmail.com
Evaluation of participants and/or data management	Site investigator	Complejo Hospitalario de Navarra, Pamplona, Spain	Lacruz, Francisco francisco.lacruz.bescos@cfnavarra.es
Coordination at the center Evaluation of participants and/or data management	Site investigator/PI	Hospital Universitario Son Espases, Palma de Mallorca, Spain	Legarda, Inés ines.legarda@ssib.es
Evaluation of participants and/or data management	Site investigator/PI	Complejo Hospitalario de Toledo, Toledo, Spain	López Ariztegui, Nuria nlariztegui@gmail.com
Evaluation of participants and/or data management	Site investigator	Institut d’Assistència Sanitària (IAS)—Instituí Cátala de la Salud. Girona, Spain	López Domínguez, Daniel
Coordination at the center Evaluation of participants and/or data management	Site investigator/PI	Hospital La Princesa, Madrid, Spain	López Manzanares, Lydia lydialopez@hotmail.com
Evaluation of participants and/or data management	Site investigator	Hospital Universitario Clínico San Carlos, Madrid, Spain	López Valdés, E evalopezvaldes@yahoo.es
Evaluation of participants and/or data management	Site investigator	Hospital La Princesa, Madrid, Spain	Lorenzo Barreto, Pablo pablorenzobarreto@gmail.com
Evaluation of participants and/or data management	Site investigator	Hospital Universitario Vall d’Hebron, Barcelona, Spain	Lucas del Pozo, Sara
Evaluation of participants and/or data management	Site investigator	Hospital Universitario Virgen del Rocío, Sevilla, Spain	Luque Ambrosiani, Antonio Cristobal
Evaluation of participants and/or data management	Site investigator	Hospital Universitario Virgen del Rocío, Sevilla, Spain	Macías García, Daniel dmacias-ibis@us.es
Evaluation of participants and/or data management	Site investigator	Fundación Hospital de Alcorcón, Madrid, Spain	Macías, Yolanda ymacias@cop.es
Evaluation of participants and/or data management	Site investigator	Hospital Universitari de Tortosa Verge de la Cinta, Tortosa, Tarragona, Spain	Mandrá, Marcos Alfredo
Evaluation of participants and/or data management	Site investigator	Hospital Infanta Sofía, Madrid, Spain	Mata, Marina mmataal@yahoo.es
Coordination at the center Evaluation of participants and/or data management	Site investigator/PI	Hospital Universitario Ramón y Cajal, Madrid, Spain	Martínez Castrillo, Juan Carlos jcmcastrillo@gmail.com
Coordination at the center Evaluation of participants and/or data management	Site investigator/PI	Hospital Universitario Central de Asturias, Oviedo, Spain	Menéndez González, Manuel manuelmenendez@gmail.com
Coordination at the center Evaluation of participants and/or data management	Site investigator/PI	Hospital Universitario Virgen del Rocío, Sevilla, Spain	Mir, Pablo pmir@us.es
Evaluation of participants and/or data management	Site investigator	Complejo Asistencial Universitario de Burgos, Burgos, Spain	Miranda Santiago, Javier miranda.medicina@gmail.com
Evaluation of participants and/or data management	Site investigator	Complejo Hospitalario de Toledo, Toledo, Spain.	Morales Casado, Maria Isabel mabelmc3006@gmail.com
Evaluation of participants and/or data management	Site investigator	Hospital Universitario Virgen del Rocío, Sevilla, Spain	Muñoz Delgado, Laura lmunoz-ibis@us.es
Evaluation of participants and/or data management	Site investigator (from MAR/23)	Hospital La Princesa, Madrid, Spain	Muro García, Inés ines.murog@gmail.com
Evaluation of participants and/or data management	Site investigator	Hospital Universitario Puerta de Hierro, Madrid, Spain.	Novo Ponte, Sabela snovoponte@gmail.com
Evaluation of participants and/or data management	Site investigator	Hospital Universitario Virgen del Rocío, Sevilla, Spain	Ojeda Lepe, Elena
Evaluation of participants and/or data management	Site Investigator/PI	Hospital Rey Juan Carlos, Madrid, Spain, Madrid, Spain.	Ordás, Carlos carlos.ordas@quironsalud.es
Evaluation of participants and/or data management	Site investigator	Hospital Universitario Ramón y Cajal, Madrid, Spain	Pareés, Isabel isabel.parees@salud.madrid.org
Evaluation of participants and/or data management	Site Investigator	Hospital de Sant Pau, Barcelona, Spain	Pascual-Sedano, Berta BPascual@santpau.cat
Evaluation of participants and/or data management	Site Investigator	Complex Hospitalari Universitari Moisés Broggi, Sant Joan Despí, Barcelona, Spain.	Peral Quirós, Alejandro
Evaluation of participants and/or data management	Site investigator	Hospital Universitario Virgen del Rocío, Sevilla, Spain	Pérez Calvo, Cristina
Evaluation of participants and/or data management	Site investigator	Hospital Universitario Virgen Macarena, Sevilla, Spain	Pérez Noguera, Rafael rafapereznoguera@gmail.com
Evaluation of participants and/or data management	Site investigator	Complex Hospitalari Universitari Moisés Broggi, Sant Joan Despí, Barcelona, Spain.	Planas-Ballvé, Ana
Evaluation of participants and/or data management	Site investigator/PI (since JAN/2026)	Hospital Universitari Mutua de Terrassa, Terrassa, Barcelona, Spain	Pons Pons, Gracia Gràcia Pons Pons
Evaluation of participants and/or data management	Site investigator	Institut d’Assistència Sanitària (IAS)—Instituí Cátala de la Salud. Girona, Spain	Prats, Marian Ángeles mpratssedano@gmail.com
Evaluation of participants and/or data management	Site Investigator	Hospital de Sant Pau, Barcelona, Spain	Puig-Daví, Arnau apuigd@santpau.cat
Coordination at the center Evaluation of participants and/or data management	Site Investigator/PI	Complejo Hospitalario Universitario de Ferrol (CHUF), Ferrol, A Coruña, Spain	Puy Núñez, A Alfredo.Puy.Nunez@sergas.es
Evaluation of participants and/or data management	Site Investigator	Hospital Clínico San Carlos, Madrid, Spain	Ribacoba, Carmen carmenribacoba@gmail.com
Evaluation of participants and/or data management	Site Investigator	Hospital Universitario Marqués de Valdecilla, Santander, Spain	Rivera Sánchez, María mariajesus.rivera@scsalud.es
Evaluation of participants and/or data management	Site investigator	Hospital General Universitario de Elche, Elche, Spain	Rodríguez Pérez, Amparo Belén
Neuroimaging studies	Site investigator	Hospital Universitario Virgen del Rocío, Sevilla, Spain	Roldán, Florinda florivict@gmail.com
Evaluation of participants and/or data management	Site investigator	Fundación Hospital de Alcorcón, Madrid, Spain	Roson, Mirian Mirian.roson@salud.madrid.org
Evaluation of participants and/or data management	Site investigator/PI	Hospital Universitario Donostia, San Sebastián, Spain	Ruíz Martínez, Javier javier.ruizmartinez@osakidetza.eus
Evaluation of participants and/or data management	Site investigator	Hospital Universitario Virgen del Rocío, Sevilla, Spain	San Eufrasio Martínez, Manuela
Study coordinator and data management	Site investigator	Hospital Universitario Virgen del Rocío, Sevilla, Spain	Sánchez Martín, María Elena
Evaluation of participants and/or data management	Site investigator/PI	Hospital Universitario Puerta de Hierro, Madrid, Spain	Sánchez Alonso, Pilar pisanchezal@gmail.com
Evaluation of participants and/or data management	Site investigator	Hospital Universitario Ramón y Cajal, Madrid, Spain	Sánchez Díez, Gema
Management, dissemination and collaboration	Site investigator	Hospital Universitario 12 de Octubre, Madrid, Spain	Sánchez Ferro, Álvaro alvarosferro@hotmail.com
Evaluation of participants and/or data management	Site investigator	Hospital Universitario Marqués de Valdecilla, Santander, Spain	Sánchez Peláez, Maria Victoria victoria.sanchez@scsalud.es
Coordination of the COPPADIS-2015	Coordinator of the Project	CHUAC, Complejo Hospitalario Universitario de A Coruña	Santos García, Diego diegosangar@yahoo.es
Coordination at the center Evaluation of participants and/or data management	Site investigator/PI	Complejo Hospitalario Universitario de Pontevedra (CHOP), Pontevedra, Spain	Seijo, Manuel manuel.seijo.martinez@sergas.es
Evaluation of participants and/or data management	Site investigator	Hospital Universitario Marqués de Valdecilla, Santander, Spain	Sierra Peña, María maryasyerra@hotmail.com
Coordination at the center Evaluation of participants and/or data management	Site investigator/PI	Institut d’Assistència Sanitària (IAS)—Instituí Cátala de la Salud. Girona, Spain	Solano, Berta berta_solano@hotmail.com
Review and monitoring database	Study coordinator	CHUAC, Complejo Hospitalario Universitario de A Coruña	Solleiro Vidal, Ángela angela.solleiro@outlook.es
Neuropsychologist; evaluation of participants	Site investigator	Hospital Universitario Central de Asturias, Oviedo, Spain	Solares, Carmen carmen.solares@ispasturias.es
Neuropsychologist; evaluation of participants	Site investigator	Complejo Hospitalario Universitario de Pontevedra (CHOP), Pontevedra, Spain	Suárez Fuentes, Nora
Evaluation of participants and/or data management	Site investigator	Complejo Hospitalario de Toledo, Toledo, Spain	Tabar Comellas, Guillermo
Evaluation of participants and/or data management	Site investigator	Hospital Universitario Virgen Macarena, Sevilla, Spain	Torres Moral, Alejandro alextorresmoral1995@gmail.com
Evaluation of participants and/or data management	Site investigator/PI	Hospital Arnau de Vilanova, Valencia, Spain	Valero, Caridad carivalero@icloud.com
Evaluation of participants and/or data management	Site investigator	Hospital Universitario Son Espases, Palma de Mallorca, Spain	Valero García, María Fuensanta mariafuensanta.valerogarcia@ssib.es
Coordination at the center Evaluation of participants and/or data management	Site investigator/PI	Fundación Hospital de Alcorcón, Madrid, Spain	Vela, Lydia lvela61@gmail.com
Evaluation of participants and/or data management	Site investigator	Hospital Universitario Son Espases, Palma de Mallorca, Spain	Vives Pastor, Bárbara barbara.vives@ssib.es

## References

[1] Badenoch J.B., Paris A., Jacobs B.M., Noyce A.J., Marshall C.R., Waters S. (2024). Neuroanatomical and prognostic associations of depression in Parkinson’s disease. J. Neurol. Neurosurg. Psychiatry.

[2] Prange S., Klinger H., Laurencin C., Danaila T., Thobois S. (2022). Depression in Patients with Parkinson’s Disease: Current Understanding of its Neurobiology and Implications for Treatment. Drugs Aging.

[3] Liu D., Qi C., Huang J., Xie H., Zhuang Y., Zhang Q., Zhao X., Hu T., Qin G., Lu Y. (2026). Predicting Long-Term Depression Progression in Parkinson’s Disease: A Machine-Learning Survival Analysis and Risk Score. CNS Neurosci. Ther..

[4] Wang Z., Wei H., Xin Y., Qin W. (2025). Advances in the study of depression and anxiety in Parkinson’s disease: A review. Medicine.

[5] Santos-García D., De Deus F.T., Cores B.C., Valdés A.L., Suárez C.E., Aneiros Á., Jesús S., Aguilar M., Pastor P., Planellas L. (2021). Mood in Parkinson’s disease: From early- to late-stage disease. Int. J. Geriatr. Psychiatry.

[6] Rohde C., Langeskov-Christensen M., Jørgensen L.B., Borghammer P., Østergaard S.D. (2025). Depression preceding and following the diagnosis of Parkinson’s disease and Lewy body dementia. Gen. Psychiatry.

[7] Leentjens A.F., Pontone G.M. (2026). Perspective: Depression in Persons with Parkinson’s Disease. Mov. Disord..

[8] Folkerts A.-K., Nielsen J., Gollan R., Lansu A., Solfronk D., Monsef I., Ernst M., Skoetz N., Zeuner K.E., Kalbe E. (2023). Physical Exercise as a Potential Treatment for Fatigue in Parkinson’s Disease? A Systematic Review and Meta-Analysis of Pharmacological and Non-Pharmacological Interventions. J. Park. Dis..

[9] Santos García D., de Deus Fonticoba T., Suárez Castro E., Borrué C., Mata M., Solano Vila B., Cots Foraster A., Álvarez Sauco M., Rodríguez Pérez A.B., Vela L. (2019). Non-motor symptoms burden, mood, and gait problems are the most significant factors contributing to a poor quality of life in non-demented Parkinson’s disease patients: Results from the COPPADIS Study Cohort. Park. Relat. Disord..

[10] García D.S., Fonticoba T.d.D., Cores C., Muñoz G., González J.M.P., Miró C.M., Suárez E., Jesús S., Aguilar M., Pastor P. (2021). Predictors of clinically significant quality of life impairment in Parkinson’s disease. npj Park. Dis..

[11] Santos-García D., Fonticoba T.d.D., Castro E.S., Díaz A.A., Bartolomé C.C., Panceiras M.F., González J.P., Aymerich L.V., Moreno J.G., Estrada M.B. (2020). Quality of life and non-motor symptoms in Parkinson’s disease patients with subthreshold depression. J. Neurol. Sci..

[12] García D.S., Jesús S., Aguilar M., Planellas L.L., Caldentey J.G., Caballol N., Legarda I., Vara J.H., Cabo I., Manzanares L.L. (2019). COPPADIS-2015 (COhort of Patients with PArkinson’s DIsease in Spain, 2015): An ongoing global Parkinson’s disease project about disease progression with more than 1000 subjects included. Results from the baseline evaluation. Eur. J. Neurol..

[13] Santos-García D., Mir P., Cubo E., Vela L., Rodríguez-Oroz M.C., Martí M.J., Arbelo J.M., Infante J., Kulisevsky J., Martínez-Martín P. (2016). COPPADIS-2015 (COhort of Patients with PArkinson’s DIsease in Spain, 2015), a global—Clinical evaluations, serum biomarkers, genetic studies and neuroimaging—Prospective, multicenter, non-interventional, long-term study on Parkinson’s disease progression. BMC Neurol..

[14] Beck A.T., Steer R.A., Brown G.K. (1996). Beck Depression Inventory.

[15] American Psychiatric Association (1994). Diagnostic and Statical Manual of Mental Disorders.

[16] Judd L.L., Rapaport M.H., Paulus M.P., Brown J.L. (1994). Subsyndromal symptomatic depression: A new mood disorder?. J. Clin. Psychiatry.

[17] Jenkinson C., Fitzpatrick R., Peto V., Greenhall R., Hyman N. (1997). The Parkinson´s Disease Questionnaire (PDQ-39): Development and validation of a Parkinson´s disease summary index score. Age Ageing.

[18] Santos García D., de la Fuente-Fernández R. (2013). Impact of non-motor symptoms on health-related and perceived quality of life in Parkinson’s disease. J. Neurol. Sci..

[19] da Rocha N.S., Power M.J., Bushnell D.M., Fleck M.P. (2012). The EUROHIS-QOL 8-item index: Comparative psychometric properties to its parent WHOQOL-BREF. Value Health.

[20] Reijnders J.S., Ehrt U., Weber W.E., Aarsland D., Leentjens A.F. (2008). A systematic review of prevalence studies of depression in Parkinson’s disease. Mov. Disord..

[21] Cong S., Xiang C., Zhang S., Zhang T., Wang H., Cong S. (2022). Prevalence and clinical aspects of depression in Parkinson’s disease: A systematic review and meta-analysis of 129 studies. Neurosci. Biobehav. Rev..

[22] Chendo I., Silva C., Duarte G.S., Prada L., Vian J., Quintão A., Voon V., Ferreira J.J. (2022). Frequency of Depressive Disorders in Parkinson’s Disease: A Systematic Review and Meta-Analysis. J. Park. Dis..

[23] Qin Y., Li J., Quan W., Song J., Xu J., Chen J. (2024). Risk of Parkinson’s disease and depression severity in different populations: A two-sample Mendelian randomization analysis. Brain Behav..

[24] Bega D., Luo S., Fernandez H., Chou K., Aminoff M., Parashos S., Walker H., Russell D.S., Christine C.W., Dhall R. (2015). Impact of Depression on Progression of Impairment and Disability in Early Parkinson’s Disease. Mov. Disord. Clin. Pract..

[25] Su W., Liu H., Jiang Y., Li S., Jin Y., Yan C., Chen H. (2021). Correlation between depression and quality of life in patients with Parkinson’s disease. Clin. Neurol. Neurosurg..

[26] Pontone G.M., Bakker C.C., Chen S., Mari Z., Marsh L., Rabins P.V., Williams J.R., Bassett S.S. (2016). The longitudinal impact of depression on disability in Parkinson disease. Int. J. Geriatr. Psychiatry.

[27] Sujith P., Arjunan P., Iype T., Natarajan V. (2023). Depression in Patients With Parkinson’s Disease: A Hospital-Based Cross-Sectional Study. Cureus.

[28] Dissanayaka N.N., Sellbach A., Silburn P.A., O’Sullivan J.D., Marsh R., Mellick G.D. (2011). Factors associated with depression in Parkinson’s disease. J. Affect. Disord..

[29] Camerucci E., Lyons K.E., Pahwa R. (2024). Predicting Depression in Parkinson’s Disease Using Commonly Available PD Questionnaires. J. Clin. Med..

[30] Lord S., Galna B., Coleman S., Burn D., Rochester L. (2013). Mild depressive symptoms are associated with gait impairment in early Parkinson’s disease. Mov. Disord..

[31] Saragoussi D., Christensen M.C., Hammer-Helmich L., Rive B., Touya M., Haro J.M. (2018). Long-term follow-up on health-related quality of life in major depressive disorder: A 2-year European cohort study. Neuropsychiatr. Dis. Treat..

[32] Huang Y. (2026). A review of neurophysiological relationships between sleep disorders and depression. Brain Behav. Immun. Health.

[33] Samizadeh M.-A., Fallah H., Toomarisahzabi M., Rezaei F., Rahimi-Danesh M., Akhondzadeh S., Vaseghi S. (2023). Parkinson’s Disease: A Narrative Review on Potential Molecular Mechanisms of Sleep Disturbances, REM Behavior Disorder, and Melatonin. Brain Sci..

[34] Zhu S., Li Y., Fang P., Wang T., Liu L., Li Y., Bi K., Ding S., Zhu J., Zhu D. (2026). Reduced sleep efficiency leads to cognitive dysfunction in major depressive disorder via disrupting fronto-visual circuit functional connectivity. J. Affect. Disord..

[35] Fang H., Tu S., Sheng J., Shao A. (2019). Depression in sleep disturbance: A review on a bidirectional relationship, mechanisms and treatment. J. Cell. Mol. Med..

[36] Burchill E., Watson C.J., Fanshawe J.B., Badenoch J.B., Rengasamy E., Ghanem D.A., Holle C., Conti I., Sadeq M.A., Saini A. (2024). The impact of psychiatric comorbidity on Parkinson’s disease outcomes: A systematic review and meta-analysis. Lancet Reg. Health Eur..

[37] Schrag A. (2006). Quality of life and depression in Parkinson’s disease. J. Neurol. Sci..

[38] Lacy B., Piotrowski H.J., Dewey R.B., Husain M.M. (2022). Severity of depressive and motor symptoms impacts quality of life in Parkinson’s disease patients at an academic movement clinic: A cross-sectional study. Clin. Park. Relat. Disord..

[39] Li J., You J., Li Z., Zang J., Wu L., Zhao T. (2025). Progress and prospects of Parkinson’s disease with depression research: A global bibliometric analysis based on CiteSpace. Medicine.

[40] Bock M.A., Brown E.G., Zhang L., Tanner C. (2022). Association of Motor and Nonmotor Symptoms With Health-Related Quality of Life in a Large Online Cohort of People With Parkinson Disease. Neurology.

[41] Girges C., Vijiaratnam N., King A., Auld G., McComish R., Chowdhury K., Ambler G., Maclagan K., Limousin P., Athauda D. (2026). Mild-to-moderate depressive symptoms impact on self-reported outcome measures in clinical trials for neurodegenerative diseases. Clin. Trials.

[42] Stark C., Beck J., Oswald A., Rogausch A., Schreiner A.-K., Cody R., Hohberg V., Knappe F., Kreppke J.-N., Ludyga S. (2026). Lifestyle physical activity coaching in outpatients with major depressive disorder (PACOUTPAT): Study protocol for a randomized controlled trial on physical activity, depression, and quality of life. Trials.

[43] Sahu S., Singh S., Tripathi R.K., Tripathi S.M., Nischal A. (2026). A Cross-sectional Case-control Study on Metacognition, Neurocognitive Functions, and Quality of Life Among Older Adults with Depressive Disorder. Indian J. Psychol. Med..

